# Understand me: Youth with chronic pain on how knowledge gaps influence their pain experience

**DOI:** 10.1080/24740527.2022.2146489

**Published:** 2023-01-26

**Authors:** Cara L. Brown, Gayle Restall, Francis Austin S. Diaz, Polina Anang, Kerstin Gerhold, Heidi Pylypjuk, Kristy Wittmeier

**Affiliations:** a Department of Occupational Therapy, College of Rehabilitation Sciences, Rady Faculty of Health Sciences, University of Manitoba, Winnipeg, Manitoba, Canada; b Rady Faculty of Health Sciences, Max Rady College of Medicine, University of Manitoba, Winnipeg, Manitoba, Canada; c Department of Psychiatry, Max Rady College of Medicine, Rady Faculty of Health Sciences, University of Manitoba, Winnipeg, Manitoba, Canada; d Children’s Research Institute of Manitoba, University of Manitoba, Winnipeg, Manitoba, Canada; e Rady Faculty of Health Sciences, University of Manitoba, Winnipeg, Manitoba, Canada; f Department of Pediatrics and Child Health, Rady Faculty of Health Sciences, University of Manitoba, Winnipeg, Manitoba, Canada; g Mississippi Center for Advanced Medicine, Mississippi, USA

**Keywords:** chronic pain, youth, qualitative, resources, patient engagement

## Abstract

**Background:**

There is a perceived lack of readily available resources to support self-management skills in youth living with chronic pain. The perspectives of youth regarding information gaps may improve the effectiveness of resources developed for them.

**Aim:**

The aim of this study was to explore the perspectives of youth living with chronic pain on the interactions among their pain experiences, chronic pain resources and research.

**Methods:**

Using an interpretive paradigm, we interviewed seven participants (age range 12–19 years) diagnosed with chronic pain. Two frameworks for meaningful engagement of citizens in research and policy informed the interview guide. Data were analyzed inductively using content analysis approaches to examine patterns and develop themes.

**Results:**

The participants’ perceptions were captured by the overarching theme of “understand me.” Four subthemes elaborate on the relationship between the participants’ experiences and how their lives could be enhanced through research and knowledge mobilization. In the subtheme “my unique pain experience,” the participants help us understand them by chronicling the variation in presentation of their chronic pain. The subtheme “people don’t know it’s a thing” emphasizes that there is general misunderstanding of chronic pain by the public and in the participants’ support systems. The first two subthemes influence the third, which describes how the pain “kind of stops you from living.” The fourth subtheme, “knowledge offers hope,” offers a solution to dismantling misunderstanding of youth living with chronic pain.

**Conclusion:**

Future work needs to focus on embedding health literacy and knowledge mobilization into health and education structures to promote developmentally relevant self-management skills.

## RÉSUMÉ

**Contexte**: Il y a un manque perçu de ressources facilement disponibles pour soutenir les compétences d’auto-prise en charge chez les jeunes vivant avec de la douleur chronique. Les points de vue des jeunes sur les lacunes en matière d’information peuvent améliorer l’efficacité des ressources mises au point pour eux.

**Objectif**: Le but de cette étude était d’explorer les perspectives des jeunes vivant avec de la douleur chronique sur les interactions entre leurs expériences de douleur, leurs ressources de douleur chronique et leurs recherches.

**Méthodes**: À l’aide d’un paradigme d’interprétation, nous avons interrogé sept participants (âgés de 12 à 19 ans) qui avaient reçu un diagnostic de douleur chronique. Deux cadres pour une participation significative des citoyens à la recherche et aux politiques ont éclairé le guide d’entrevue. Les données ont été analysées par induction à l’aide d’approches d’analyse du contenu afin d’examiner les modèles et développer des thèmes.

**Résultats**: Le thème général de « comprenez-moi » reflète bien les perceptions des participants. Quatre sous-thèmes approfondissent la relation entre les expériences des participants et la façon dont leur vie pourrait être améliorée par la recherche et la mobilisation des connaissances. Dans le sous-thème « mon expérience de douleur unique », les participants nous aident à les comprendre en racontant les variations dans la façon dont leur douleur chronique se présente. Le sous-thème « les gens ne savent pas que ça existe » souligne qu’il y a une incompréhension générale de la douleur chronique par le public et dans les systèmes de soutien des participants. Les deux premiers sous-thèmes influencent le troisième, qui décrit comment la douleur « vous empêche de vivre en quelque sorte. » Le quatrième sous-thème, « les connaissances apportent de l’espoir », offre une solution pour démanteler l’incompréhension des jeunes vivant avec la douleur chronique.

**Conclusion**: Les travaux futurs doivent se concentrer sur l’intégration de la littératie en santé et de la mobilisation des connaissances dans les structures de santé et d’éducation afin de promouvoir des compétences d’auto-prise en charge pertinentes sur le plan du développement.

## Introduction

Chronic pain is a common condition in adolescents, with approximately 1 in 20 adolescents living with moderate to severe chronic pain.^1^ Chronic pain can negatively impact participation in school and leisure activities and family and peer relationships. It is associated with mental health concerns such as depressive symptoms^2^ that have a pervasive impact on adolescents during a critical developmental period.^3,4^ The best evidence for chronic pain treatment for adolescents promotes the use of a biopsychosocial model delivered by an interdisciplinary team^5^ that includes self-management. In self-management approaches, intervention moves “beyond education, to teaching individuals to actively identify challenges and solve problems associated with their illness.”(pe25)^6,7^ Self-management incorporates the tasks of problem solving, decision making, resource utilization, partnerships with health care providers, and taking action for medical, behavioral, and emotional management. A self-management approach can support adolescents working to develop health-related behaviors that they can carry with them throughout their life span. Because self-management is influenced by the individual’s context, including their family, community, and health services,^6^ considering this context is important in intervention. Therefore, it is important for adolescents, their families, communities, and health service providers to have resources that support developmentally appropriate self-management approaches over the course of their developmental journey to adulthood.

There is a perceived lack of readily available and accessible resources on chronic pain to support a self-management approach with adolescents.^8^ The Partnering for Pain project reported on the results of a robust priority setting process that included 215 clinicians and people with lived experience with pediatric pain as patients or caregivers. Included in the top ten priorities was the development of strategies to increase awareness, knowledge, recognition, and understanding of pain among health care providers and educators.^8^ Three years later, MacKenzie and colleagues conducted a needs assessment regarding evidence about children’s pain that included patients, families and caregivers, researchers, and other knowledge users (health care providers, educators, policymakers).^9^ MacKenzie’s team reported findings similar to those from the Partnering for Pain project.^9^ All participant groups in the needs assessment experienced barriers to evidence, although the barriers they cited were different, highlighting the importance of tailoring resources to different groups of people.^9^ The top priorities for pediatric pain management according to the patients and family members were the development of resources for patients and caregivers, the engagement of patients and caregivers in developing resources, and increased awareness about pain.^9^

To properly address the priority of knowledge mobilization for chronic pain, patient and caregiver involvement is required. Understanding the experience of adolescents and their perspectives regarding information gaps may improve the design, content, credibility, uptake, and effectiveness of resources targeting them.^10–12^ Resources that have been informed by those with lived experience may also be useful to parents, health care providers, and educators. There has been some previous co-development work with adolescents and young adults who experience chronic pain. Stinson and colleagues used information from qualitative interviews to determine the topics required in an internet-based self-management program.^4^ Slater and colleagues engaged 15- to 24-year-olds to test acceptability and usability of online resources,^13^ including the one developed from the work by Stinson and colleagues. Twiddy and colleagues interviewed young adults to determine assessment and rehabilitation needs.^3^ Collectively, these studies emphasized the importance of meaningful engagement in designing and sharing developmentally appropriate resources because adolescents and young adults are experiencing a unique time of life where one typically becomes more independent from their guardians.^3,4^ Resources that resonate with the intended users of the information in content, presentation, marketing, and mobilization platform is required.^13^

Our work focused on the involvement of people 10 to 19 years old to improve resource development and mobilization for this age group and their guardians, friends, teachers, health care providers, and other social supports. The World Health Organization defines this age range as “adolescents”^14^; however, we use the term “youth” herein because this is the term we used in recruiting and interacting with the participant group described in this study. To advance understanding of how to improve the development and mobilization of resources for youth living with chronic pain, we conducted a youth-engaged study in which youth reviewed and critiqued existing chronic pain resources in a series of workshops. Prior to the first workshop, the youth were interviewed about their experiences, expectations, and perspectives to inform the workshop planning and evaluation. This article reports on the perspectives of the youth as expressed in these interviews about the interactions among their pain experiences, chronic pain resources, and research.

## Methods

### Context of the Study

**Figure 1.**
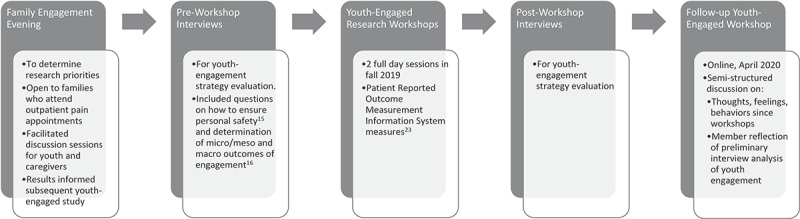
Overview of the multiphase study. This study reports on data collected in phase 2, pre-workshop interviews.

This article report on one part of a multi-stage study (see Figure 1). The first phase was a local half-day research priority-setting event that was attended by youth living with chronic pain and their families. During the event, three main priorities were identified: (1) the importance of the youths’ perspectives on chronic pain, (2) a need for improved access to resources, and (3) the value of peer-to-peer interaction. Subsequently, we developed a youth-engaged workshop series that brought youth participants with lived experience of chronic pain together to review and critique a selection of chronic pain resources. We were interested in both the outcomes of the workshop as well as understanding how well we engaged the youth. One data collection strategy to evaluate the youth engagement was interviews that were conducted with the youth before and after the workshop series. During the pre-workshop interviews, participants provided rich data on their experiences in relation to resources, information, and research on chronic pain. This article reports on an analysis of these pre-workshop interviews.

### Conceptual Framework

Our multiphase study was guided by two frameworks that promote meaningful engagement of citizens in research and policy development. The first was a trauma-informed intersectional analysis described by Shimmin and colleagues.^15^ This approach seeks to rebalance the power between researcher and “patient” by situating “patient engagement” to promote health equity and social justice. This approach acknowledges that both the researchers and the “patients” are complex intersectional beings who have experiences that shape their interactions, some of which may have been traumatic. This approach encouraged our research team to design research processes that would not perpetuate existing health inequities. Our processes to promote a trauma-informed approach included (but were not limited to) the following:

- Inviting researchers to draw on all their perspectives, both professional and personal.
- Ensuring that the interviewer was knowledgeable in the use of a trauma-informed approach.
- Informing the participants before the interview and workshop series that their role was of “youth researcher.”
- Using a meaningful member-checking approach that included an individual living with chronic pain on the analytic team.
- Continual discussion and reflexivity on power, privilege, and safety.

The second framework was a validated conceptual outcomes framework for citizen-user involvement.^16^ This framework emphasizes the importance of understanding and promoting positive outcomes of engagement processes that are intended to improved health services (including resource development) and policy. The framework encourages exploration and evaluation of the effects of engagement on multidimensional individual and social structures. Positive outcomes of engagement at micro, meso, and macro levels increases accountability and promotes positive sociopolitical and organizational outcomes while avoiding harm to the individuals participating.^16^ In this study, we kept these considerations in mind as we developed the study and its evaluation. Examples of ways in which we incorporated these concepts were creating processes that supported the individual perspectives of the youth about their involvement through pre- and post-workshop interviews, designing age-appropriate and engaging activities during the workshop and monitoring participants’ responses so that the goals of the research to develop high-quality resources could be achieved, ensuring that the voices of the participants would be reflected in knowledge translation materials that could influence future engagement, and encouraging active and meaningful participation to promote the potential for participants to be willing to engage in future research.

### Design

We used an interpretive paradigm.^17^ This approach acknowledges the contextual experience of the youth being interviewed and accepts the possibility of multiple realities.^18,19^ Specifically, we used interpretive description, a qualitative approach that acknowledges the theoretical and practical knowledge that researchers bring to a project.^18,19^ With this paradigm, researchers seek to be transparent in how their perspectives and the participant perspectives are co-constructing the results.^20^ The study was approved by the University of Manitoba’s Human Research Ethics Board (H2018:417; HS22261).

### Setting

The researchers and youth in this study all live in a Canadian prairie province that does not have a formal pediatric pain clinic. At the time of the study, the province had a single pediatric pain specialist (K.G.) who provided outpatient appointments once a week for children and teens with chronic pain at an urban tertiary care center.^21^ Physical and occupational therapy was available for consultation with families upon physician request, and youth were referred to psychology and psychiatry as indicated. One barrier to accessing the pediatric pain specialist was the geographical dispersion of the population in this province; another was a waitlist with quickly growing wait times that resulted in a median wait of over one month to first visit.^21^

### Participants

Recruitment information for the workshop series was posted in the outpatient pediatric pain specialist waiting area, and study information was available on the provincial pain strategy website.^22^ In addition, participants of the previous half-day priority setting meeting were notified about the workshop series. We aimed to recruit six to eight youth between the ages of 14 and 18 who had current or recent (within the past 3 years) lived experience with chronic pain. The age criteria were expanded (to as young as 12) to recruit our target of six to eight people for the workshops. One participant was 18 at the time of recruitment but age 19 at the time of the pre-workshop interview. Though it was a not a part of the inclusion criteria, all participants were receiving or had received services from the outpatient consultative pain service described above. An exclusion criterion was being medically unstable at the time of recruitment. All participants provided written informed consent in person prior to data collection; for youth under the age of 18, a parent or guardian also provided written consent.

### Data Collection

To describe the participants, we collected information using the Patient Reported Outcome Measurement Information System (PROMIS)^23^ prior to the workshops and a brief demographic questionnaire that was sent to participants after participation in the workshops. PROMIS^23^ includes measures for anxiety, depression, fatigue, pain interference, pain behavior, and peer relationships. The demographic questionnaire included questions on gender, ethnicity, and whether the individual self-identified as being part of a racialized group. Because of the potential for identification of individuals based on the demographic data, we present the demographic results in generalities.

Qualitative data collection consisted of semistructured interviews prior to the workshops. All youth who participated in the workshop series also participated in a pre-workshop interview. The interviews were conducted in a private room in a research institution physically linked to the medical center where the outpatient pediatric pain specialist is located. Three of the interviews were conducted over the phone due to geographical barriers to in-person interviews. Youth were encouraged to be interviewed without a guardian present, but the youth’s needs guided whether a guardian was present in the interview space. A guardian was present in one of the in-person interviews, and in one phone interview it became apparent that a guardian was physically present, when the guardian came on the phone midway through the interview to explain that the participant needed a moment to collect themselves. Otherwise, no dialogue or interaction between the participants and guardians was noted.

**Figure 2.**
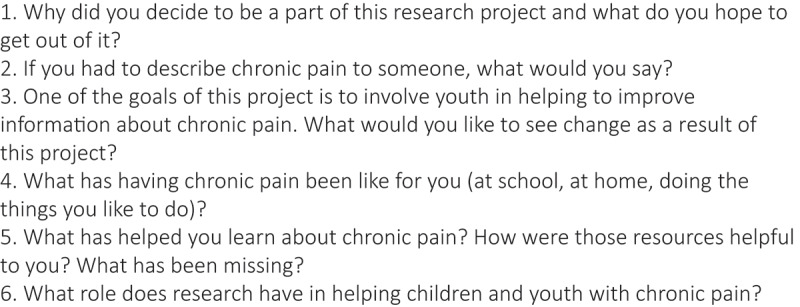
Interview questions.

The semistructured interview guide was originally designed for planning and evaluation of the workshop series youth researcher engagement strategy. See Figure 2 for interview questions. The development of the interview content was informed by the two frameworks previously described above. The intersectional, trauma-informed approach to engagement framework guided the development of questions that asked input from the youth researchers on how to create a safe space for their engagement.^15^ The conceptual outcomes framework for citizen-users guided the development of questions that could be used to determine the outcomes of involving the youth researchers at micro (personal), meso (organizational), and macro (wider societal) levels.^16^ The questions for the pre-workshop interviews focused on (1) the participant’s understanding of what chronic pain is, (2) the participant’s personal experiences with chronic pain, (3) how research can help people with chronic pain, (4) the participant’s motivation and expectations for the workshops, and (5) how the participant’s comfort could be supported in the workshops. See Figure 1 for the interview questions.

The interviews were conducted by F.D., who is experienced in conducting qualitative interviewing. We did not pilot the interview guide. The interviews were conducted over a period of several weeks, and F.D. engaged in reflection between interviews to consider changes to prompts for subsequent interviews. F.D. was a registered occupational therapist at the time of the interviews and thus was able to attend to any emotional distress that may have been expressed by participants. All interviews were audio-recorded and transcribed verbatim. Pseudonyms were used in place of participant names for confidentiality.

### Data Analysis

The PROMIS^23^ questionnaires were analyzed using the HealthMeasures Scoring System, which converts data into standardized *T*-scores (mean = 50, SD = 10) and assigns descriptions to score ranges (e.g., within normal limits, mild, moderate, severe) to assist with interpretation.^23,24^ The reference populations for the Anxiety, Depression, Fatigue, Pain Interference, and Peer Relationships subscales include the general population and children who live with pain, and the reference population for pain behavior is children who live with pain.^23^

The qualitative analysis was primarily conducted by C.B., F.D., G.R., and K.W. The researchers acknowledge that their positionality and worldviews influenced the research process, and they engaged in reflective discussion to make their assumptions explicit and consider how underlying biases may be influencing their interpretation. C.B., K.W., and G.R. are White settler clinician researchers who were trained in Euro-Western research methodologies.^17^ C.B. and G.R. are registered occupational therapists without any affiliation with the youth pain outpatient services. K.W. is a registered physical therapist who works with youth, including clinical consultation for outpatient chronic pain that the study participants attended or had attended in the past. F.D. was a licensed occupational therapist with experience working with youth with disabilities and was a first-year medical student at the time of interviews and analysis. The analysis team also gained medical clinical perspectives from K.G. and P.A. and consumer/health care provider perspectives from H.P. and a young adult with personal experience of chronic pain who reviewed the interview transcripts and contributed to insights and interpretation of the data.

Analysis took an iterative approach. Our first approach to the data included the entire data set; that is, both pre- and post-workshop interviews. This was done with the objective of evaluating the youth engagement strategy. All researchers on the analysis team read through all of the transcripts to get an understanding of the whole of the data. Data were first analyzed by K.W. and F.D. independently using manifest content analysis^26^ by developing descriptive codes inductively. The interview guide was used to as a tool to maintain focus on the youth engagement evaluation objective.^15,16^ Next, codes were grouped into categories to condense the data into a description that retained the original meaning of the data. These steps were completed using Microsoft Word software. The analysis team met to engage in reflexive discussion about the descriptive themes being developed and started to consider interpretive, or latent, understandings of the data.^26^ Themes were compiled in a written summary that was verbally presented with main points on a PowerPoint slide to four of the seven participants in an online meeting (the third of three study workshops as depicted in Figure 1). This third workshop had been moved online due to public health orders related to increasing rates of COVID-19 in the region. The four participants who were in attendance provided feedback and additional data related to these themes that informed subsequent analysis.

During this initial approach to the data, we recognized that the pre-workshop interviews contained a substantial amount of rich data about participants’ experiences that we had not anticipated collecting. These data provided a deeper understanding of the relationship of the youths’ experiences to their views about knowledge and research that had relevance for chronic pain resource development and design beyond our initial objective of evaluating our public engagement strategy. Therefore, we conducted additional analysis using only the pre-workshop interviews. These contained the most data on personal experiences and an understanding of the youths’ perspectives prior the workshop series. To add this rich data to the already developed coded data set, C.B. inductively coded the transcribed interviews focusing on the participants’ experiences. This was done using the qualitative analysis software NVivo (12 Plus)^25^ to store, organize, and code the data. As codes to capture experiences were developed, codes were added, combined, or collapsed with the codes developed in the first phases of analysis to describe the story of the youths’ perspectives. C.B. used a latent approach, looking at the intention of what was said^26^ to develop themes that represented the main constructs in the data and their relationships with one another. File and reference counts of codes helped C.B. understand the extent to which different codes and themes represented the experience of this group of participants as a whole. K.W., C.B., and G.R. engaged in in-depth discussion of the findings, with K.W. and G.R. challenging the assumptions of C.B. and contributing to identification of links between the data to ensure that the findings were true to the youth voices. The final stage of analysis involved engagement of a young adult living with chronic pain who reviewed and provided feedback on the final analysis.

Trustworthiness in our analytic process was enhanced by inviting participants to engage in reflection on the preliminary results after the first stage of analysis, incorporating the interpretation of a young adult researcher who reviewed the raw and themed data, incorporating multiple researchers, and balancing the perspectives of people with lived experience (youth participants, young adult researcher, clinicians who provide pain services) with more outsider lenses (F.D., G.R., C.B.).

## Results

The seven youth participants were aged 12 to 19. Five participants resided within the province’s major urban center and two resided in other communities. Pain duration ranged from 3 months to 5 years (median 3 years). Mean participant PROMIS scores fell within the severe category for anxiety, depression, and fatigue; moderate for pain interference; mild for pain behavior; and fair for peer relationships. There were seven responses to the survey on gender and ethnicity. The participants responding to the survey identified as female or demi-girl and reported having European origins or being Canadian. None of the survey respondents identified as being a racial minority. Two of the responses were identical; because the survey was anonymous, there is no way to know whether one participant responded twice.

The youths’ voices provided insight into their lived experiences and contexts and how they felt their lives could be enhanced through research and knowledge mobilization. Although we began the project by addressing an important clinical question about youth perspectives of existing chronic pain resource material, the youth provided rich insight into their lived experiences and contexts and how they felt their lives could be improved through research and knowledge mobilization. The results of our study reflect these insights and their interconnectness.

An overarching theme of “understand me” illustrated how the participants felt that chronic pain and their experience of living with chronic pain are not understood by society. The participants perceived that both research and knowledge mobilization can touch on their lives positively in many ways. The participants thought that enhanced knowledge about chronic pain would improve their quality of life and that research gives credibility to their experience. Angie captured this idea of how a lack of knowledge of chronic pain is directly related to people’s lack of understanding of her as a person and how knowledge could support her when asked what could be done to improve her situation.

More people to understand that chronic pain actually exists. Like, it’s a, not like just in your head, make up kind of thing. Like, I’ve had teachers … just saying that I’m pretending, that they don’t believe in chronic pain. So maybe just getting more awareness and, I know, you probably won’t be able to fix it per se, but, like, at least trying to find better ways to treat it. Or, like, better, like meditation or different exercises that you can do to help lessen the pain. (And the awareness needs to be for) mainly the general public but also some doctors, because when I first, like, started feeling the pain, they were thinking that, after they did, like, neuro tests and there was nothing physically wrong, they were just saying that I was lying.

**Figure 3.**
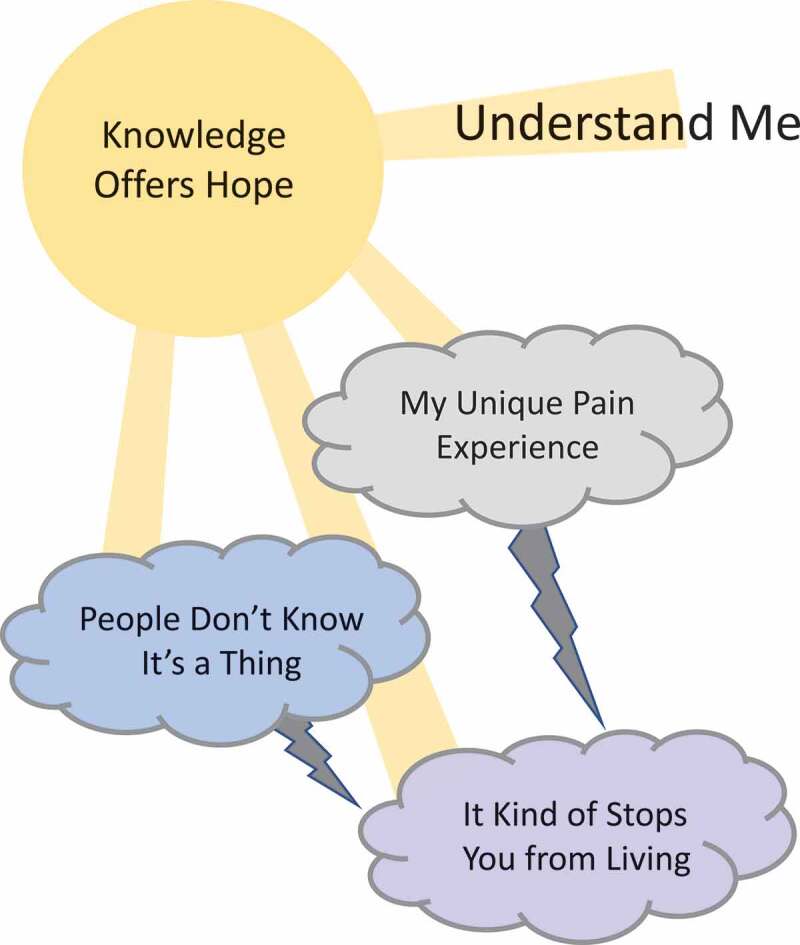
Conceptual representation of the results to show primary relationships between themes. “Understand Me” is the overarching theme representing how the participants’ primary desire is to be better understood. The storm clouds indicate themes that conceptualize the challenges the participants face in their daily lives with chronic pain. The thunder bolts represent how some of the experiences exacerbate others. The theme “knowledge offers hope” is represented as sunshine that can positively impact the youths’ experiences and ultimately result in the youth feeling better understood.

We chronicled the complexity of the participants’ experience in four interconnected themes and two subthemes. Two of the themes primarily focus on the youths’ unique experiences of living with pain and how it impacts their lives overall (“my unique pain experience,” “it kind of stops you from living”). The theme of “it kind of stops you from living” also includes two subthemes, “strained relationships” and “emotional toll,” because these are two negative outcomes of the participants’ attempts to balance their participation in their life with taking care of themselves. The third theme, “people don’t know it’s a thing,” focuses on the pervasive lack of understanding of chronic pain by the youths’ social circles and the public in general that negatively impacts the youths’ lived experience. The fourth theme, “knowledge offers hope,” focuses on how research and knowledge can positively impact the previous three themes and ultimately help youth be and feel more understood. See Table 1 for a listing of the themes, subthemes, and major codes and Figure 3 for an image that illustrates the relationships between the themes.

**Table 1.** Themes, subthemes, codes, and subcodes within overarching theme “understand me.”

Theme (subthemes)	Code (subcode) examples
My unique pain experience	Everybody’s pain is different; my pain (temporal, quality, location, etc.); lack of control
It kind of stops you from living (strained relationships; emotional toll)	Impact on participation (school, leisure, chores, etc.); “normal”; impact on relationships (friends, teachers, parents, etc.); impact on mental health
People don’t know it’s a thing	Lack of knowledge (parents, teachers, friends, etc.); misconceptions; hard to understand; hard to explain; invisible
Knowledge offers hope	Research priorities; helpful resources; positive features of resources; resource gaps

### My Unique Pain Experience

All participants emphasized how every person with chronic pain has a unique experience and felt that the individualized experience and expression of chronic pain contributes to misunderstanding of the condition. In the interviews, participants were asked to describe what chronic pain is, from their perspective. Most participants were unable to provide a generic definition; instead, they explained that the experience of chronic pain is unique in every individual. Devonne stated,

It kind of looks different on everybody. Because some people are lying in bed unable to get up. They can’t eat or they’re in the hospital having IVs given into them. And some people are out playing soccer, basketball. But they’re still going through the same thing. It’s just sometimes it’s harder on some people.

Though we did not ask the participants to explain their personal pain experience, most of the youth did so in order explain what chronic pain is and how it presents differently for each individual. These rich descriptions seemed to be one way in which the participants were trying to be better understood. The participants’ rich descriptions of their own pain, taken together, vividly illustrated their point that everyone with chronic pain has different pain quality, location, and intensity experiences. Pain quality ranged from feeling like burning (Angie) to “a knife twisting just constantly. Sometimes it’s faster. Sometimes it’s slower. Sometimes the knife is sharper. Sometimes it’s duller. Kind of just depends on the day” (Carly). Variation was also described in relation to the location of pain; Gracie experienced the pain in her back and joints, whereas Carly’s pain was in her upper abdomen. Brooke and Angie both had pain that spread from an initial location over time. Devonne said, “For me it’s throughout my whole body. So, it’s a pain every single day. It just moves around my body. One day it could be in my head, one day my knee, one day my stomach, one day my shoulder.” Regarding temporality, Brooke was the only individual with pain-free periods. The other participants used the words “constant” and “never-ending” to describe their pain but had fluctuations in the intensity of the pain.

Some participants who had lived with pain longer also described what they have learned about their individual triggers for pain, which revealed an understanding of a link between their physical symptoms and their emotional state. However, variation also was evident with triggers; for example, Brooke and Carly noted that their pain increased in intensity with anxiety and stress, and Carly learned over time that her pain increased in large social groups. Not all participants had this level of understanding—some of the participants had less insight into their pain triggers and were struggling to understand the “cause” of their pain.

Participants believed that the variation in presentation of chronic pain from person to person is why people have trouble understanding chronic pain and that this makes developing educational resources challenging. When Carly imagined trying to create a resource to explain chronic pain, she said, “So it’s hard to talk about generalized symptoms. What to look for? You know like you talk about the flu. There are about 12 symptoms that you can almost guarantee come with that. … It’s harder with chronic pain because it’s so personal.”

### “People Don’t Know It’s a Thing”

All participants expressed frustration with the lack of knowledge that people have about chronic pain and, in particular, chronic pain in children and youth. Participants felt that the knowledge gap is so wide that some people do not even know that chronic pain is a legitimate medical condition or, as Brooke said, “People don’t know it’s a thing.” This lack of knowledge leads to misconceptions about chronic pain and the people experiencing it. Participants also thought that lack of a known physical cause contributed to a lack of understanding. Angie said, “It’s real that, um, I’m not making it up and, uh, don’t really know what caused, like, started it. But just because it’s not something physical doesn’t mean that it doesn’t actually exist.”

Several participants in this study indicated that they were still trying to understand chronic pain and what underlies it, and other participants said their journey to understanding had been long and winding. Most participants did not feel that they got adequate information from their physicians except for a few participants who felt well educated by their chronic pain specialist or family physician. According to the participants, general practice physicians need more training in chronic pain or need to learn how to better express their knowledge, because several participants perceived that the doctors did not know enough about chronic pain to diagnose it, explain it, or treat it. When explaining how she was diagnosed, Francine said, “Well, eventually my mom asked my doctor if it was the chronic illness that I have. And, um, my doctor had to look it up and realized, yea, that’s probably it.” And Carly felt that her doctor had little to offer her in terms of education, “So, it’s like, that’s interesting that I took, I don’t know, an hour, an hour and a half of my private Googling to figure out a little more than my doctor even knew.”

A major misconception arising from the lack of information in society about chronic pain was that the pain experience is not real. Participants had been questioned by family, friends, and teachers who thought they were “faking it” or thought that the participants should just “get over it.” Participants felt that they were being accused of using their chronic pain diagnosis as an excuse to avoid undesirable activities. Though participants were frustrated by the lack of knowledge in the public about chronic pain, they also acknowledged that chronic pain is a difficult condition to explain. They said that even when it was explained well, pain can be difficult for people who do not experience pain to understand. However, they still saw ways to address this issue; Carly pointed out that there are other complex conditions that, with advocacy or social marketing, have become more accepted, like multiple sclerosis.

A few participants thought that the invisible nature of the disease contributed to misunderstanding. “I get that they don’t understand because chronic pain isn’t something that you can see” (Grace). When participants stay home because they are not well, people only see them when they “look fine.” This resulted in people not understanding fully how the chronic pain affected the participants. Carly explained,

They [teachers and friends] saw me happy. They saw me doing schoolwork and then all of a sudden I was gone for a week. So they don’t quite see that I’m doubled over in bed screaming or that I’m popping all these pain pills or that I’m in the ER. Like, they don’t witness that. So it’s understandable why they don’t believe necessarily that’s real because they see that I’m perfectly fine and then I’m gone and then I’m perfectly fine again.

Participants felt the burden of needing to explain chronic pain to the people around them in order to be understood. Doing this education was difficult for participants because of the general lack of knowledge in the public about chronic pain, the complexity of the condition, the lack of resources to help describe chronic pain in simple terms, and society’s idea of what a “real” medical condition is. Further, even with attempts to explain their situation, most participants experienced people thinking that they were faking their pain experience, thus devaluing the participants and their experiences.

### “It Kind of Stops You from Living”

This theme speaks to the complex interplay between the physical and emotional symptoms outlined in the theme “my unique pain” and the social context described in the theme “people don’t know it’s a thing” and how they interact to interfere with desired adolescent lives. Two subthemes (“strained relationships” and “emotional toll”) reflect negative outcomes that resulted from participants’ attempts to balance their participation in typical youth activities with attempts to manage their chronic pain. Eloise captured the sentiment of life disruption when she said, “[The pain], it’s pretty constant and is very fast to stop your life sort of. Like, it kind of stops you from living sometimes … waking up every day and knowing that there’s going to be limited things you can really do.”

Several participants often used the term “normal” when describing their challenges with participation, saying they were not able to do “normal” things or that sometimes they tried to act “normal.” This idea of “normalcy” seemed to be related to multiple factors, including not being able to do all of the activities that they wanted to do, not being able to do the activities that they expected they would be doing at this point in their lives, not being able to keep up with peers in activities, and needing to moderate or hide their negative emotional state around other people.

In the following statement, Francine referred to the abrupt change in her activities because of the physical and emotional impact of chronic pain, “Going from being healthy and going from someone who has chronic illness because things like school and activities and hobbies and stuff change in an instant. And it’s kind of hard to go through and not have so many things in your life anymore.”

The participants gave up a lot of leisure and extracurricular activities like dancing and soccer because of symptoms or as a coping mechanism to save energy because they used a lot of energy to participate in school. Even participation in leisure activities that could be less physically demanding like playing musical instruments, writing, drawing, playing video games, and unstructured socializing were affected, despite creative attempts to continue with these activities. Angie explained how she tried to adapt her leisure activity of music to her limitations related to chronic pain:

I play clarinet as well. I used to play trumpet, which I love playing the trumpet, but the vibrations of the mouthpiece on my face would make my face burn, so I couldn’t play it anymore. So I had to move to clarinet. And even then, sometimes the fingering of it makes me burn.

The pain also interfered with the participants’ abilities to contribute to age-appropriate productive activities, like household chores and engaging in paid work. For example, Angie wanted to help with the grocery shopping, but she had to avoid pushing a shopping cart because the vibration from the handle triggered her pain. Devonne quit a job at a day care because it was too difficult to be in a caregiving role while having chronic pain. The participants had to make difficult decisions about what activities to stop doing to save energy for more essential activities such as school.

Participation in school was an emotionally charged topic and a challenge for all participants. Some of the challenges included participating fully in physical education class, being distracted from tasks by pain sensations, and drowsiness as a side effect from medications. Being unable to fully participate in school had a cascading effect on the participants’ lives. It impeded their relationships with their teachers and affected their self-esteem when their grades were negatively impacted. Several participants also emphasized that school was a major link to their social circle and that not participating in school obstructed their ability to stay connected with their social circles. Francine said: “So things like friendship goes away, and attendance is also very affected. And once your attendance goes down, everything else collapses. … So things like your grades and your friends and everything connected to school will just feel completely lost.”

#### Strained Relationships

Strained relationships was one of the major consequences of chronic pain that was discussed by the youth. The youth spoke of having difficulty with developing and maintaining trusting and authentic relationships with parents, friends, medical professionals, and teachers. On this topic, the link between a lack of knowledge, feeling misunderstood, and how it impeded daily life was explicit in the participants’ stories. Teachers were the main focus of the participants’ talk related to relationships. Participants said that teachers questioned the validity of their pain when their class participation was inconsistent. Grace expressed frustration with needing to repeatedly explain her activity limitations:

And you always have to explain to a teacher, and … you shouldn’t have to feel the need to do that. But then they question you and you feel—you feel that like where is the trust? Like don’t you not, do you not trust me to—to feel like I can sit down without having someone question me?

The combination of missed classes and participants feeling the need to defend themselves made youth–teacher relationships particularly difficult. Carly explained how her absenteeism influenced how the teachers perceived her:

Because of that [diagnosis], I had missed a lot of school in my 11th and 12th year of high school, which resulted in some of my teachers treating me very poorly. I was told by numerous teachers to my face that I wasn’t graduating even if I had the grades I needed because they didn’t like my attitude.

These challenging relationships interfered with the youth seeing their teachers as role models or mentors. None of the youth talked about any teachers who were a positive support in their life.

Difficulties participating in school also fractured relationships with friends at a time in life when friendships are very important developmentally for transitioning from childhood to young adulthood. Carly described what happened when she stopped attending school regularly:

My friends essentially cut me off from communication. It was a matter of out of sight, out of mind, so because I missed so much, even though we had many different ways of communication even though I wasn’t in school, which I don’t blame them for, I just wasn’t there.

Friendships were challenging because the participants felt that they needed to continually explain their limitations. Devonne said, “With my friends, they forget a lot of the time if I have chronic pain, and they’ll just want to do normal stuff with me that I can’t participate in because of it. And then I’ll have to tell them all over again.” Grace described how the challenging relationships with friends intersected with feeling misunderstood:

It’s been hard because they, they just want to be there for you. Like my friends just want to be there for me and they want to, they want to understand what I’m going through. But obviously they can’t. And I just feel that with—it’s been difficult for them to understand. It’s been frustrating for me because I want them to understand.

The interruption of the processes of building supportive relationships with peers was common among all participants. There was a slightly different pattern for family relationships, with more variation in the participants’ experiences with family relationships, but all participants’ family relationships had features that were atypical of an adolescent trajectory. Even when the youth felt supported by their family, the youth experienced family-related stress because they felt like they were a burden on their family and tried to protect their family members from their negative experiences. The participants’ stories around these relationships also linked to knowledge, because the participants perceived that their parents were lacking knowledge of how to help their child deal with their pain experience. Angie said, “And like it’s also, I think, like, a big drain on my family when I’m feeling pain. Like, sometimes I won’t tell them that I’m in pain because I just see that it makes them so upset because they feel helpless.”

#### Emotional Toll

The second major consequence of living with chronic pain in daily life for these participants was the emotional toll. Not only did the participants need to cope with physical symptoms but they also needed to actively figure out how to adapt their participation day-to-day and constantly explain their needs for accommodation while experiencing these physical symptoms.

Angie’s statement provides a glimpse into this when she said:

People with chronic pain are trying their best to like function normally, but it takes a toll on you, not only, like, the pain but mentally where it’s like … where you just wake up in the morning when you’re in pain and then you go to bed when you’re in pain. And, like, that’s not physically exhausting but also mentally draining.

Many participants also described anxieties that took up their energy like wondering if there was a sinister cause to their pain that was being missed or worry about being able to keep up with school because of absences. An example of this is when Eloise said,

So any time I feel any sort of pain from my body I, uh, immediately think that it’s actually something else instead. So since my pain is extremely constant, I’m always wondering what’s wrong with me. And if I’m going to die soon or something like that.

Several youth referred to the challenges of living with chronic pain for several years and how this made it difficult for them to maintain a positive outlook and maintain their mental health. Grace said,

It’s been really hard over the—the past two years. I’ve had difficulty with being able to—to see the bright side of things. … You can’t always see the good side of things. You always have to fight extra hard to be able to see like the light on the other side of the tunnel because if you don’t, you get trapped to this dark place and you can’t get out of it.

This comment by Grace about having trouble staying positive was echoed by other participants and related to how their chronic pain impacted their self-concept. Their chronic pain persona of being irritable, tired, and negative did not align with their self-concept of who they really were and who they wanted to be. This was another area where the idea of “normalcy” arose and links together the ideas of emotional toll and relationship strain. The participants sometimes felt like they needed to act “normal” around other people. Putting on this act was exhausting. Sometimes participants chose to sit out of activities to avoid needing to put on this mask of “normalcy.” Devonne’s story illustrated this:

And if I decide to act normal, then I have to stay like that the whole time, and it’s very energy draining. So it’s nice to have some friends that I can just, you know, relax around. I don’t have to worry about that. But the friends that I have to keep a front and stay, like, happy and energetic and, like, non limping, even though I limp, like, less limp. And, like, no ice packs or anything for a certain amount of time. I try to stay away from those friends because it’s very draining on me.

### Knowledge Offers Hope

This theme describes an alternative outcome that the participants envisioned with improved research, knowledge, and resources on chronic pain for youth and their family, friends, teachers, doctors, and the public. In this alternative outcome, youth would feel understood, connected, supported, and empowered while living with chronic pain.

The few participants who had spent time reading about chronic pain and one who felt well educated by a pain specialist spoke of the positive impact of this information on their quality of life. These participants wished they had had this information sooner and hoped that other youth would not have to go for a long time without this information like they did.

[The information] helps everyone have a better understanding about what it is and what these kids are going through. So then people aren’t just trying to guess or assume. Then it’s actually research. It’s actually knowledgeable. You can look back on it. It’s credible. And the youth will feel relieved that, hey, look, there’s—this is exactly what I’m going through. Like, that’s what I want. So then they won’t feel alone. They don’t feel misread or understood. (Devonne)

Though participants spoke more about being able to access and relate to information, they also valued research as important for positively impacting youth with chronic pain. They understood that research might help with learning about the cause of the condition and, subsequently, how to treat it. They felt that the most important contribution of research on chronic pain was how it could validate chronic pain experiences. They spoke of how research on the condition means it is a “real” condition and that youth who have chronic pain are worth investing in. Carly said,

[Research gives] something to grasp onto. … It just gives you something to look forward to, to hope for. It makes you feel like somebody cares about what’s happening. If nobody’s researching it, that means it’s not a problem. People look to find problems. People research things to find a problem. If nobody’s researching anything, if nobody’s asking you questions about how to make anything better, it really means, at least to me, that nobody cares. Research means somebody cares.

This idea of research giving credibility to youths’ pain experiences ties back onto the overarching theme of “understand me.” Participants wanted people to recognize and understand what they are going through. This included having health care providers easily recognize and provide information about their condition. Participants also wanted peers and teachers to be more knowledgeable about chronic pain: its individual presentation, its variability, and its fluctuating nature. This would help participants to manage their participation in activities with the support of their friends and teachers without having their motivations misunderstood and having to explain themselves. For youth to feel understood and validated and to improve their quality of life, they felt that knowledge gaps need to be bridged through formal research as well as resources.

## Discussion

The objective of this article was to report on the perspectives of youth on the interactions between their pain experiences, chronic pain resources, and research in their everyday lives. The results of our study highlight how the participants perceived that there is inadequate knowledge of chronic pain and that this lack of knowledge creates misconceptions about youth living with chronic pain that ultimately affects quality of life. The results of this study provide insights for clinicians, researchers, and policymakers about how to improve the experience of youth through consideration of developmental context in knowledge mobilization for adolescents with chronic pain and their support circles.

Our findings support and extend previous literature on this topic. The stories of the participants in this study illustrate findings reported in quantitative studies. For example, previous research has described how youth with chronic pain miss school, withdraw from activities, and tend to be overdependent on parents.^27^ A qualitative study by Forgeron and colleagues,^28^ who interviewed 16 adolescents between 14 and 19 years of age about their friendships, also found that adolescents with chronic pain are left feeling misunderstood by their friends. Forgeron and colleagues suggested that misunderstandings stem from adolescents struggling to explain their condition because they only have a superficial understanding of it, as well as the public’s lack of understanding of the difference between acute and chronic pain.^28^ Our article adds more depth in understanding how youth relate a lack of knowledge of chronic pain in their social circles to their quality of life. In particular, our study adds to knowledge on the perception of youth regarding the knowledge gaps of their teachers and physicians. The youth believed that more knowledge would reduce the daily social emotional work that they engage in to maintain belonging despite needing to participate in different ways. To understand how to tailor resources for teachers and families to help them support youth with chronic pain, we can look to work by Riggenbach and colleagues,^29^ who argued for the use of self-determination theory for supporting individuals with pediatric chronic pain. Self-determination theory posits that there are three primary psychological needs: autonomy, competence, and relatedness. Autonomy is when the youth feel they have personal choice and a sense of freedom over their actions. In an autonomy-supported context, the people around the individual support independence at a “just right” level, where the person does not feel left on their own to manage by themselves but is satisfied with the amount of independence they have. Because our participants with chronic pain perceived that teachers and peers did not have enough knowledge or information to understand their situation, they did not feel like they were in an autonomy-supportive context. Consistent with other studies, we found that youth can be stigmatized by everyone in their social circles (including teachers, parents, and peers) and that the root of stigma is a lack of knowledge.^28,30^

Despite previous work on developing resources for youth, our findings suggest that there are major gaps in knowledge mobilization for this age group, because several participants, despite having and living with a firm diagnosis of chronic pain, indicated that they had very little knowledge of the condition. Others felt that they had worked very hard on their own to access resources that helped them understand chronic pain. The Canadian Pain Task Force found that “people living with chronic pain want more to be done to increase self-education about pain management and more opportunities to share their experiences with and help others also living with pain.”^31^ Youth are no exception. Developmentally contextual knowledge mobilization is important for this age group because of their unique developmental needs. Youth today are looking for resources that combine multiple needs, such as the need for accessible and reliable information, as well as social support and connection.^10^

Youth in this study particularly emphasized school and relationships at school as a source of stress. In line with an autonomy-supporting environment, the youth were looking for a school environment where the teachers understand their condition and work together with them to develop appropriate accommodations, without the youth needing to repeatedly explain their circumstance or be questioned about their motivations. This finding is immediately relevant for clinicians and researchers, in that there is a need for knowledge mobilization that targets educators. These resources can use an autonomy-supportive framework to promote understanding of common challenges of youth living with chronic pain and how to develop reasonable individualized accommodation plans in the educational system. Integration of health and educational systems to promote the development of these individualized plans is important. Some of this work has been started, for example, by the Solutions for Kids in Pain national knowledge mobilization network,^32^ but there is still a gap between resources specific to youth audiences and their identified needs. For example, resources are needed for youth on how to communicate about their pain and for sharing with peers, because youth struggle to know how to communicate and have others understand their condition. Research on knowledge mobilization that supports youth living with chronic pain to feel empowered in sharing information to support their relationships is needed because youth living with chronic pain are inextricably connected to their social structures. Learning to develop and maintain healthy social relationships while living with chronic pain is very important for youth development.^3^

The demonstrated need for more easily accessible and youth-tailored resources reflects the intersections between self-management, health literacy, and knowledge mobilization. An individual’s ability to engage in self-management practices requires a degree of health literacy. Core dimensions of health literacy models include information seeking, defined as the ability to seek and access information, as well as the ability to process and apply information.^33,34^ A recognized limitation of health literacy conceptualizations is that the onus is largely on the individual to seek, understand, and use health-related information.^33,34^ This may explain why the participants in this study had low self-reported knowledge of chronic pain and felt that chronic pain resources were scarce and inaccessible to them. Health literacy responsiveness is a concept that seeks to balance the responsibility for health information access and use. A health literacy responsive organization or entity actively considers and embeds health literacy promoting practices into its organization or structure.^35–37^ In child and youth models of health literacy, the need for age appropriateness, cultural relevance, and social support is emphasized, which matches well with a youth’s needs for support in learning self-efficacy in self-management, just as they require it for the development of other skills.^33^ These concepts align with those underpinning knowledge mobilization; for example, ensuring that resources are tailored to the intended audience, are based on evidence (including lived experience and traditional knowledge), and are informed by people with lived experience. Participants of our study endorsed the importance of these concepts in supporting their living with chronic pain. Though the participants in this study were in a context with a lack of comprehensive interprofessional chronic pain rehabilitation program, other literature suggests that our findings are not unique to our study context.^28^ There is important work emerging to meet the unique developmental needs of adolescents and their knowledge mobilization needs, such as work looking at peer support models and the use of social media for promoting self-management skills.^38,39^ Future research also needs to move from an individual to a structural approach to knowledge mobilization and health literacy to understand how to embed knowledge mobilization in health and education systems to provide autonomy-supporting environments.

This study has limitations. This study represented the perspectives of youth who were registered for a workshop series in a particular clinical setting and may not be transferable to other youth or settings. Importantly, there was a lack of gender, racial, and ethnic diversity within the participant group. There are many possible explanations for this participant make-up. Recruitment was through the pediatric pain outpatient specialist, which is not a formally funded and advertised service. Health services are historically less accessible to oppressed racial and ethnic groups.^40^ Further, Blacks, Indigenous peoples, and people of color may not be interested in participating in research in a White Western-dominated context. Because pain is known to be experienced and expressed differently according to sex, gender, and ethnicity,^2,41^ future research needs to ensure that these groups are given voice by using anti-racist and anti-oppression methodologies and approaches.^40,42^ Another limitation is that we do not know the extent of the context of the individuals who were interviewed by phone and whether their interview context influenced their responses. Finally, the youths’ responses may have been influenced by the fact that they were embarking on participating in a workshop series with the researchers at the time of the interview. These youth may have been more interested in research and knowledge than the average adolescent living with chronic pain, and they may have had a desire to engage or respond in a way that they thought would please the researchers/workshop facilitators.

In conclusion, the female and demi-girl nonracialized youth interviewed for this study highlight that there is a general lack of knowledge about chronic pain, and this lack of knowledge meant that they were not understood, adding to the stress of living with chronic pain. The findings of this article chronicle their experiences and stories to support their desire to have their voices elevated, be heard, and be better understood. Further, we have outlined how these results point to the need for future research and clinical focus on embedding health literacy and knowledge mobilization into health and education structures to improve access to knowledge for the youth and their entire network to promote greater quality of life. Next steps include working with youth with pain and their social circles to understand how to improve knowledge mobilization regarding adolescent chronic pain to support developmentally appropriate growth of self-management skills in youth with chronic pain.
